# Munro's Reply

**Published:** 1855-04

**Authors:** 


					﻿AIR CHAMBERS.
Mr. Editor :—As I am not entirely decided in my own
mind, in relation to the propriety of air chambers for retain-
ing upper sets of teeth. I should be glad that you, or some of
your contributors would favor us with some animadversions
upon the subject in the next number of the Register. In my
practice, I have made perhaps about an equal number of each
kind, that is with and without air chambers. Some of my
very most successful cases were without chambers ; and such
adaptation of the plate, as does not require it to contain a
chamber, or to be very wide in the arch of the mouth, seems to
me a desideratum devoutly to be wished for. And yet when
I observe that nearly all the upper sets, made by other dentists,
which have lately come under my observation, contain cham-
bers, I feel distrustful of my own predilection for plates with-
out them. In the use of air chambers, I have for some time
past been very much governed by the depth of the alveolar ridge,
and the favorableness of the antagonism of the opposite teeth.
When there is sufficient depth of process combined with favo-
rable antagonism, I dispense with chambers. When an oppo-
site condition of process, and opposing teeth exists I generally
use them, proportionate in size with the relative condition of
the month. According to my experience, plates containing
chambers frequently adhere better for a short time after worn
than subsequently, while many plates without chambers con-
tinue to improve in their adhesive quality for some time after
worn.
Am 1 mistaken in the conclusion I have come to, from what
I have seen of the work of others, that most dentists at the
present time use air-chambers in nearly all their plates for
upper sets? What is your own practice in this respect, at
the present time, and why ? Has a narrow plate without any
chamber that adheres well, any advantages over a wide plate
covering the whole roof of the mouth, with a chamber setting
down from it, that adheres equally well.
Salem O, Feb. 14th, 1855.	JOHN HARRIS.
In reply to the inquiry of Dr. Harris, we would say that we
use air chambers in a large majority of cases, with a tolerably
wide plate for upper sets. A plain plate which holds
sufficiently firm, we, however, regard as preferable, and when
this can be accomplished and the plate not cover much of the
palate, it is still better. It is more pleasant to the wearer not
to have the entire palate covered with a metalic substance.
The fact that plain plates increase in adhesion more than air
chamber plates can be accounted for, from the fact that unless
the chamber is very large, it fills up more or less by the soft
parts adapting themselves to the vacuum, and thus render-
ing the chamber useless. The plain plate is improved because
the adaptation becomes more perfect by use.
We are now inserting an upper set without an air chamber,
because the palital arch is almost a flat surface, and the cham-
ber would interfere with the articulation. In this case, our
plain plate adheres better than the one before used which has
a chamber.
Plain plates which do not adhere sufficiently tight, we change
to those with chambers. We scarcely ever make a chamber
other than that we get in swedging the plate, with the pattern
of the chamber on the metallic model. We regard the accu-
rate adaptation of the plate around the external alveolar bor-
der as of the greatest importance, and the plate should extend
up as high as the soft parts will permit. The better this is ac,
complished, the less will the palital arch have to be covered.
Many plates, we are satisfied, cover too much of the soft palate
and are rendered less useful by it, for the moveability of these
parts in deglution often loosen the plate, when otherwise
it would retain its place. The great desideratum in suction
plates should be not so much the force of adhesion as the sta-
bility of the plate, when pressure is made on it at different
points.
A plate which will not loose its hold by pressure upon it at
any point, and yet can be drawn from the mouth by little force
is far better than one requiring ten or fifteen pounds force to
remove it, and which will loose its hold by slight pressure on
the front teeth or on either side.—[Ed.
				

